# Identification of Pepper *CaSBP08* Gene in Defense Response Against *Phytophthora capsici* Infection

**DOI:** 10.3389/fpls.2020.00183

**Published:** 2020-02-26

**Authors:** Huai-Xia Zhang, Xiao-Hui Feng, Muhammad Ali, Jing-Hao Jin, Ai-Min Wei, Abdul Mateen Khattak, Zhen-Hui Gong

**Affiliations:** ^1^ College of Horticulture, Northwest A&F University, Yangling, China; ^2^ Tianjin Vegetable Research Center, Tianjin Academy of Agricultural Sciences, Tianjin, China; ^3^ Department of Horticulture, The University of Agriculture, Peshawar, Pakistan

**Keywords:** pepper, *Phytophthora capsici*, SBP-box family genes, *CaSBP08*, defense genes, *Nicotiana benthamiana*

## Abstract

Little information is available on the role of Squamosa promoter binding protein (SBP)-box genes in pepper plants. This family of genes is known to have transcription characteristics specific to plants and to regulate plant growth, development, stress responses, and signal transduction. To investigate their specific effects in pepper (*Capsicum annuum*), we screened pepper SBP-box family genes (*CaSBP* genes) for *Phytophthora capsici* (*P. capsici*) resistance genes using virus-induced gene silencing. *CaSBP08*, *CaSBP11*, *CaSBP12*, and *CaSBP13*, which are associated with plant defense responses against *P. capsici*, were obtained from among fifteen identified *CaSBP* genes. The function of *CaSBP08* was identified in pepper defense response against *P. capsici* infection in particular. CaSBP08 protein was localized to the nucleus. Silencing of *CaSBP08* enhanced resistance to *P. capsici* infection. Following *P. capsici* inoculation, the malondialdehyde content, peroxidase activity, and disease index percentage of the *CaSBP08*-silenced plants decreased compared to the control. Additionally, the expression levels of other defense-related genes, especially those of *CaBPR1* and *CaSAR8.2*, were more strongly induced in *CaSBP08*-silenced plants than in the control. However, *CaSBP08* overexpression in *Nicotiana benthamiana* enhanced susceptibility to *P. capsici* infection. This work provides a foundation for the further research on the role of *CaSBP* genes in plant defense responses against *P. capsici* infection.

## Introduction

Pepper (*Capsicum annuum* L.) is a valuable solanaceous crop species with high economic as well as high nutritive value worldwide. However, it is prone to various diseases, especially *Phytophthora* blight, which is caused by *Phytophthora capsici* (Jin et al., 2016), a soil-borne pathogen that can infect various tissues of pepper plants, including roots, stems, leaves, flowers, and fruits (Wang et al., 2013a). Its host range is wide, including tomato, eggplant, cucumber, watermelon, pumpkin, snap peas, and lima beans (Granke et al., 2012). To combat pathogen infection, plants have developed a range of defense mechanisms including physiological, biochemical, molecular, and cellular processes and barriers along with inducible innate immunity (Mou et al., 2017; Hou et al., 2018). Inducible innate immunity is largely regulated at the transcriptional level by the action of many transcriptional factors. Transcription factors have important functions in combating the impact of pathogens through activating or repressing the expression of defense genes (Xu et al., 2011). For example, APETALA2/Ethylene Response Factor-domain transcription factor (AP2/ERF-ORA59) directly regulates the expression of *PDF1.2* (PLANT DEFENSIN1.2) in the process of necrotrophic pathogen infection in *Arabidopsis* (Zarei et al., 2011). The basic leucine zipper (bZIP) of the *Arabidopsis TGA6* transcription factor acts together specifically with the translocation of the NONEXPRESSOR OF PATHOGENESIS-RELATED GENES1 (NPR1) ankyrin repeat protein, which activates the salicylic acid (SA) marker gene PATHOGENESIS-RELATED-1 (*PR-1*) in the *Botrytis cinerea* necrotrophic infection process (Zhang et al., 1999; Zander et al., 2010). JAMYC2 and JAMYC10 are two other MYC transcription factors that are jasmonic acid (JA)-regulated and involved in defense responses against pathogen infection through inducing *PDF1.2* gene expression in tomato (Boter et al., 2004; Lorenzo and Solano, 2005).

Squamosa promoter binding protein (SBP)-box genes are a family of plant-specific transcription factors that contain a highly conserved SBP domain (Klein et al., 1996). The domain contains about 76 amino acids residues, including two zinc fingers and one nuclear localization signal (Yamasaki et al., 2004). Klein et al. (1996) first discovered *Antirrhinum majus* SBP-box genes, identified as *AmSBP1* and *AmSBP2*, according to their capability to interact with the floral meristem identity gene SQUAMOSA promoter sequence. Subsequently, Lännenpää et al. (2004) identified SBP-box genes in another plant, *Betula pendula* (i.e., *BpSPL1*). Similarly, Hou et al. (2013) discovered *VpSBP5* in *Vitis vinifera*, and Zhang B. et al. (2017) found two others in hexaploid wheat, i.e., *TaSPL20* and *TaSPL2*1. There are many reports of SBP-box gene involvement in the development and growth of plants. However, there are few reports about its involvement in responses to biotic and abiotic stresses, especially the former (Stone et al., 2005; Wang et al., 2016; Hou et al., 2018). For example, *AtSPL14* is induced by the fungal toxin fumonisin B1, which induces programmed cell death in *Arabidopsis* (Stone et al., 2005). Transgenic *Arabidopsis* JASMONATE CARBOXYL METHYLTRANSFERASE (*AtJMT*) plants exhibit down-regulation of *AtSPL2* (At5g43270), which has a role in the JA-mediated resistance pathway (Kim et al., 2007). Tobacco *NbSPL6* is needed to develop N-mediated resistance for combating *tobacco mosaic virus*. Moreover, the ortholog *AtSPL6 Arabidopsis* gene is necessary for the Toll and Interleukin-1 Receptor Nucleotide Binding-Leucine Rich Repeat (TIR-NB-LRR) to function in mediating resistance against *Pseudomonas syringae* infection (Padmanabhan et al., 2013). Hou et al. (2013) reported that through the SA-induced systemic acquired resistance pathway, *VpSBP5* participates in regulating resistance against *Erysiphe necator* and, in grapes, through the methyl jasmonate (MeJA)-induced wound signaling pathway. *AtSPL9* interacts with jasmonate ZIM-domain (JAZ) proteins and negatively regulates the JA response as well as resistance to insects in *Arabidopsis* (Mao et al., 2017). *SPL6* functions in the endoplasmic reticulum (ER) stress response to control ER stress signaling outputs and retain equilibrium between both adaptive and death signals to determine cell fates in rice during ER stress (Wang et al., 2018). However, compared with the widespread research on SBP-box genes in model species such as *Arabidopsis*, less information is available concerning pepper SBP-box genes, especially regarding their involvement in resistance against *P. capsici*.

In our previous research, we identified fifteen SBP-box genes in pepper (i.e., *CaSBP01*, Capana01g002647; *CaSBP02*, Capana01g002832; *CaSBP03*, Capana01g003073; *CaSBP04*, Capana01g003445; *CaSBP05*, Capana02g001917; *CaSBP06*, Capana05g002237; *CaSBP07*, Capana07g001731; *CaSBP08*, CA07g17550; *CaSBP09*, CA08g03640; *CaSBP10*, Capana10g000507; *CaSBP11*, Capana10g000709; *CaSBP12*, Capana10g000886; *CaSBP13*, Capana10g002379; *CaSBP14*, Capana11g002003; and *CaSBP15*, CA11g04690), and we named them according to their chromosomal order (Zhang et al., 2016). The *CaSBP* coding sequences ranged from 336 base pair (bp) to 3024 bp in length for *CaSBP08* and *CaSBP06*, respectively, with a nuclear localization signal occurring for all gene family members except *CaSBP08*. Additionally, all the *CaSBP* genes encoded proteins with two zinc finger-like structures, i.e. C3H and C2HC, except for *CaSBP09* and *CaSBP15*, which lack the C3H zinc finger-like structures (Zhang et al., 2016). All *CaSBP* genes are induced by compatible or incompatible strains of *P. capsici*, except for *CaSBP15,* whose expression is down-regulated during *P. capsici* infection. Some *CaSBP* genes (i.e., *CaSBP11* and *CaSBP12*) may also be involved in SA and MeJA regulation mechanisms (Zhang et al., 2016). To further study the function of *CaSBP* genes in plant resistance against the pathogen *P. capsici*, *CaSBP08*, *CaSBP11*, *CaSBP12*, and *CaSBP13*, which are involved the plant defense response against *P. capsica*, were obtained from among the fifteen identified *CaSBP* genes using virus-induced gene silencing in this work. In addition, we further investigated the function of *CaSBP08* and found it to be localized to the nucleus and to play a negative regulatory role in the plant defense response against *P. capsici* infection in pepper and transgenic *Nicotiana benthamiana*. This work provides a foundation for further research on the role of pepper SBP-box genes in plant defense responses against *P. capsici* infection.

## Materials and Methods

### Plant Material and Pathogen Preparation

Pepper cultivar AA3 and the *P. capsici* strain HX-9, provided by the *Capsicum* Research Group, College of Horticulture, Northwest A&F University, P. R. China, were tested. Plants were maintained in a growth chamber at 22°C/18°C (day/night temperatures) with a 16/8-hour photoperiod. The *P. capsici* strain HX-9 was cultured in darkness at 28°C with potato dextrose agar (PDA) medium. Sporulation induction and spore release were conducted using the method described by Wang et al. (2011) with modifications. Briefly, the HX-9 strain of *P. capsici* was first cultured on PDA in a Petri dish under darkness at 28°C for five days. Then, ten approximately 0.8-cm diameter discs were cut from PDA culture plates and grown in the dark for 3 days in 90-mm Petri dishes with 15–20 mL of 2% (*w*/*v*) cleared carrot broth at a constant temperature of 28°C. The cultures were then washed twice with sterile distilled water and covered with 15–20 mL of Petri broth [KH_2_PO_4_, 0.15 g; Ca(NO_3_)_2_, 0.4 g; CaCl_2_, 0.06 g; Mg(NO_3_)_2_, 0.15 g each per 1000 mL]. These cultures were further incubated at 28°C for five more days, before being chilled for 30 minutes at 4°C to induce zoospore release, followed by a 1-hour incubation at room temperature. A hemocytometer was used to measure zoospore concentration adjusted to 1 × 10^5^ spores/mL following the method by Jin et al. (2016). Then, 5 mL of this zoospore culture was used to inoculate *CaSBP08*-silenced, control pepper plants, and transgenic *N. benthamiana* plants *via* the root-drench method as described by Wang (2013). A detached leaf inoculation assay was prepared as per the method described by Zhang et al. (2015) and maintained at 22°C/18°C (day/night temperature) under a 16/8-hour photoperiod and 60% relative humidity in a growth chamber.

### Virus-Induced Gene Silencing of SBP-Box Family Genes in Pepper

The virus-induced gene silencing (VIGS) system based on tobacco rattle virus (TRV) was used to silence the SBP-box family pepper genes, as previously reported by Wang (2013). To generate the VIGS plasmid constructs of *CaSBP* genes, fifteen 200–500 bp fragments from the corresponding SBP-box genes were amplified using gene-specific primers. Then, their specificities were assessed using NCBI Primer BLAST (**Table S1**). Using double digestion, the acquired products were cloned into the TRV2 vectors with *Bam*HI and *Kpn*I restriction enzymes (Zhang et al., 2003). Then, they were sequenced by Sangon Biotech Company (Shanghai, China). The recombined vectors, i.e., TRV2:*CaSBP01*, TRV2:*CaSBP02,* TRV2:*CaSBP03,* TRV2:*CaSBP04,* TRV2:*CaSBP05,* TRV2:*CaSBP06,* TRV2:*CaSBP07,* TRV2:*CaSBP08,* TRV2:*CaSBP09,* TRV2:*CaSBP10,* TRV2:*CaSBP11,* TRV2:*CaSBP12,* TRV2:*CaSBP13,* TRV2:*CaSBP14,* TRV2 (negative control), TRV2:*CaPDS* (phytoene desaturase, positive control), and TRV1, were transformed into *Agrobacterium tumefaciens* strain GV3101 using freeze-thaw transformation. Pepper seedlings at the two true leaves stage were used for the procedures silencing the SBP-box genes family according to the method described by Zhang et al. (2013). All the injected plants were maintained in a growth chamber set at 18°C under darkness for two days and then moved to a growth chamber with 22°C/18°C (day/night temperatures), a 16/8-hour photoperiod, and 60% relative humidity. Forty-five days after infiltration, the silencing efficiency was measured from leaf samples collected from the silenced and control plants. Then, an assay of the detached leaves was conducted as described by Zhang et al. (2015). Five milliliters of 1 × 10^5^ spores/mL zoospore culture of *P. capsici* strain HX-9 was used to inoculate the control and silenced plants by drenching roots. Lastly, the roots and leaves of control and silenced plants were collected and stored at −80°C.

### Subcellular Localization of *CaSBP08*

The *CaSBP08* coding region without a termination codon was amplified using sequence-specific primers (**Table S1**). The obtained product was cloned into the PMD-19 vector and then cloned into the pVBG2307:*GFP* vector between the *Xba*I and *Kpn*I restriction sites to produce the final pVBG2307:*CaSBP08*:*GFP* plasmid. The recombinant fusion pVBG2307:*CaSBP08*:*GFP* plasmid was sequenced by Sangon Biotech Company and then transformed into *A. tumefaciens* strain GV3101 through freeze-thaw transformation. Then, the GV3101 cells carrying the pVBG2307:*CaSBP08*:*GFP* vector and pVBG2307:*GFP* vector (used as a control) were grown overnight in Luria-Bertani (LB) medium with the proper antibiotics. Then, infiltration buffer (10 mM MES, pH 5.7, 10 mM MgCl_2_, and 200 µM acetosyringone) was used for cell suspension, which was infiltrated into *N. benthamiana* leaves with a needleless syringe (Mou et al., 2017). After being injected, the plants were grown in a chamber set at 22°C/18°C (day/night) temperatures and a 16/8-hour photoperiod for two days and then assessed under a fluorescent confocal microscope (Olympus, Tokyo, Japan) with a 488 nm excitation wavelength.

### *N. benthamiana* Transformation

The full encoding region of *CaSBP08* (336 bp) was cloned into the pVBG2307:*GFP* vector between the *Xba*I and *Kpn*I restriction sites to produce the final plasmid pVBG2307:*CaSBP08*:*GFP* for genetic transformation (**Table S1**). Overexpression lines of *CaSBP08* were achieved through tobacco leaf disc transformation with *Agrobacterium* intervention (Oh et al., 2005). Eleven lines of transgenic *N. benthamiana* plants, each with resistance to kanamycin and having the pVBG2307:*CaSBP08*:*GFP* construct were obtained. Three transgenic lines of *CaSBP08* (lines 2, 10, and 11) were randomly selected for further study. Transformation was confirmed using quantitative real-time PCR during the T2 generation (**Table S2**). T1 plants seeds were obtained from T0 regenerated plants, and T2 lines seedlings were generated on MS agar plates with 100 µg/mL kanamycin. For further analyses, T3 plants were used.

### RNA Extraction and Quantitative Real-Time PCR

Total RNA was isolated as per the procedures described by Guo et al. (2012). The first strand cDNA was synthesized using the PrimeScript Kit (Takara, Dalian, China) according to the manufacturer's instructions. The cDNA concentration was diluted to 50 ng/µL and used for quantitative real-time PCR (qRT-PCR). Then, qRT-PCR was performed in triplicate on an iCycler iQ™ Multicolor PCR Detection System (Bio-Rad, Hercules, CA, USA) with the following thermal cycling program: pre-denaturation at 95°C for 1 min followed by 40 cycles of denaturization at 95°C for 10 s, annealing at 56°C for 30 s, and extension at 72°C for 30 s. All the primer specificities for qRT-PCR were assessed using NCBI Primer BLAST (**Table S2**). Gene expression was quantified and normalized to the expression level of actin (*CaActin2*, accession no. AY572427; *Nbactin-97*, accession No. LOC109206422) (Schmittgen and Livak, 2008; Du et al., 2015; Yin et al., 2015).

### Malondialdehyde Measurement

Following inoculation with *P. capsici*, the malondialdehyde (MDA) content of the control plants and *CaSBP08*-silenced plants were measured using a colorimetric determination technique with thiobarbituric acid from Ma et al. (2013) with modifications. For this purpose, the crude enzyme used for MDA determination was extracted using 10% trichloroacetic acid (TCA). Then, 2 mL of crude enzyme extract was mixed with 5 mL of 0.5% thiobarbituric acid (TBA) reagent, boiled for 10 min, quickly cooled, and centrifuged at 5000 × *g* for 10 min. The control contained 2 mL of distilled water instead of the crude enzyme. Absorbance was measured at 600 nm, 532 nm, and 450 nm.

### Peroxidase and Catalase Activity Measurements

After inoculation with *P. capsici*, the peroxidase (POD) and catalase (CAT) activities of the *CaSBP08-*silenced and control plants were measured using the guaiacol method and ultraviolet spectrophotometry, respectively (Hammerschmidt et al., 1982). The crude enzymes, used to determine POD and CAT activity, were extracted using 0.2 M phosphate buffer (pH 7.8) and 0.05M Tris-HCl buffer (pH7.0), respectively. The POD activity determination reaction included 0.1 mL of crude enzyme, 2 mL of 0.3% H_2_O_2_, and 0.9 mL of 0.2% guaiacol. The CAT activity determination reaction included 1.0 mL of Tris-HCl, 1.7 mL of distilled water, 0.5 mL of crude enzyme, and 0.1 mL of 100 µM H_2_O_2_.

### Disease Index Percentage Statistics

Subsequent to *P. capsici* inoculation, the percent disease index values of plants were recorded following the procedure described by Zhang (2009). Sixteen days after *P. capsici* HX-9 strain inoculation, the *CaSBP08*-silenced and control plants infection symptoms were categorized into five levels: Level 0, no symptoms; Level 1, lower leaves of plants yellowing or wilting; Level 2, lower leaves of plants with obvious defoliation or whole plants wilting; Level 3, stem base black, except for new growth, with all leaves fallen; and Level 4, whole plant death. Thirteen days post-inoculation with *P. capsici*, the symptoms in the transgenic lines were also categorized into five levels: Level 0, no symptoms; Level 1, whole plant wilting, with no constriction between stems and leaves; Level 2, whole plant wilting, with death of lower leaves and constriction between stems and leaves; Level 3, all leaves dead, except those at the point of new growth, with constriction occurring between stems and leaves; Level 4: death of the whole plant. Disease index percentages were recorded based on the following formula:

$$\text{Disease index percentage}=\frac{\Sigma \text{the numerical grade of disease}\times \text{number of disease plants of this grade}}{\text{the highest grade of disease}\times \text{total number of surveys}}\times 100$$

### Statistical Analysis

Least significant difference (LSD) values were calculated using Data Processing System 7.05 (DPS 7.05, China), a software package with comprehensive experimental design and statistical analysis functions. Significance was determined at *P* ≤ 0.05 or *P* ≤ 0.01 thresholds. All the experiments, with at least three biological replicates, were conducted and evaluated separately.

## Results

### Pepper Plant *CaSBP08*, *CaSBP11*, *CaSBP12*, and *CaSBP13* Genes Are Involved in Resistance to *P. capsici*

#### Phenotypic Observation and Silencing Efficiency of *CaSBP*-Silenced Plants

**Figure 1:**
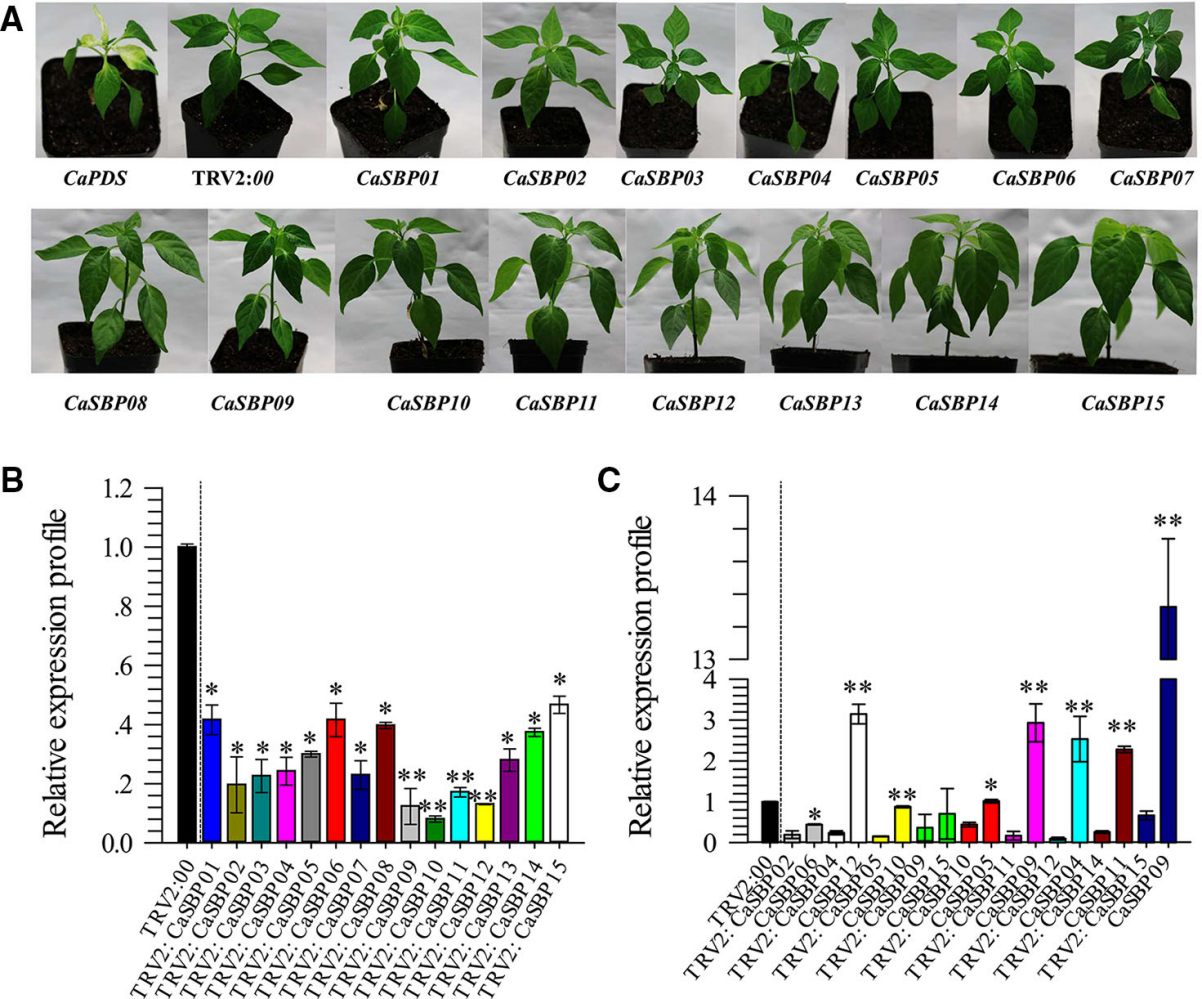
Phenotypes and silencing efficiency of the fifteen members of CaSBPs silenced plant. **(A)** Photographs were taken forty days after injection (the diameter of the pot is 7 cm). **(B)** Silencing efficiency of the fifteen members of *CaSBPs* in their corresponding silenced plants. **(C)** The expression level of the pepper SBP-box genes, which with the highest homology with their corresponding silenced gene. * and ** represent significant differences at P ≤ 0.05 and P ≤ 0.01 respectively. Mean values and SDs for three biological replicates are shown.

To screen for genes that respond to *P. capsici* infection, fifteen *CaSBP* genes were silenced using the VIGS method. In this study, the pepper *CaPDS* gene (phytoene desaturase, GenBank accession number, X68058) was taken as a positive control, which induces a leaf photo-bleaching phenotype when silenced. For the negative control, an empty TRV2:*00* vector was selected. Forty days after injection, the positive control (TRV2:*CaPDS*) plants showed photo-bleaching, while the TRV2:00 plants and plants with each of the fifteen *CaSBP* genes silenced exhibited no obvious phenotypic changes (**Figure 1A**). Subsequently, we detected the silencing efficiency of the fifteen *CaSBP* genes. As shown in **Figure 1B**, the fifteen *CaSBP* genes were silenced compared with the negative control, and the silencing efficiencies were between 50% and 90%. To ensure the silencing specificity of the target *CaSBP* genes, the expression of both the target genes as well as the genes with the highest homology to the target genes were also measured. As shown in **Figure 1C**, when the target gene was silenced, the expression levels of the *CaSBP* genes and their respective genes with the highest homology can be divided into three categories. The first category includes silenced target genes (i.e., *CaSBP02* or *CaSBP09*) for which their corresponding gene of highest homology (i.e., *CaSBP06* or *CaSBP15*) is also silenced, but with a silencing efficiency lower than that of the target gene. The second category included target genes that were silenced (i.e., *CaSBP04*, *CaSBP11*, *CaSBP12*, *CaSBP14,* or *CaSBP15*), but their corresponding highest homology genes (i.e., *CaSBP12*, *CaSBP09*, *CaSBP04* or *CaSBP09*) had increased expression. The third category includes silenced target genes (i.e., *CaSBP05* or *CaSBP10*) with corresponding highest homology genes (i.e. *CaSBP10* or *CaSBP05*) having unchanged expression. The *CaSBP* genes with the highest homology with the targeted silenced genes are shown in **Table S3**.

### Identification of Resistance Associated With *CaSBP* Genes

**Figure 2:**
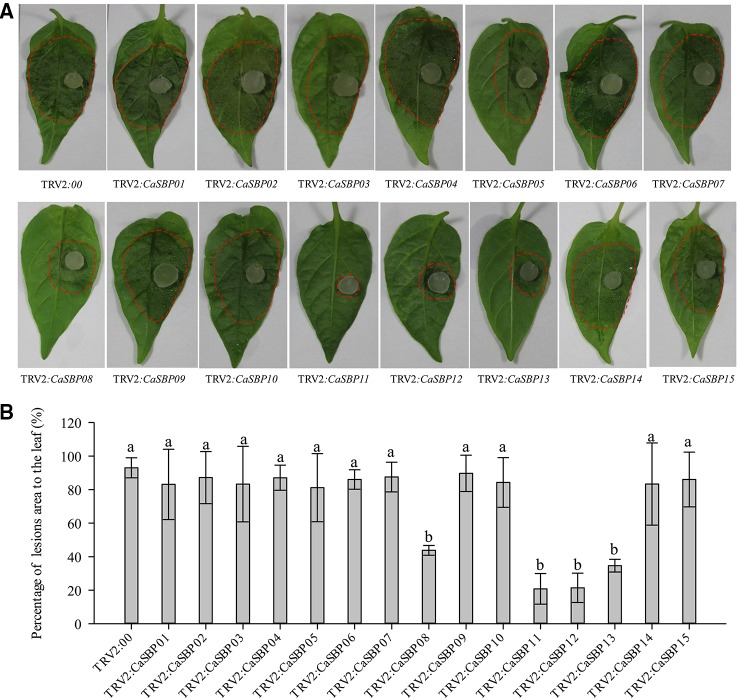
Phenotype and percentage of the lesion area of detached leaves of the fifteen members of *CaSBPs* ^,^ silenced and control plants after inoculation with *P. capsici*. **(A)** Phenotype of detached leaves of the fifteen members of *CaSBPs* ^,^ silenced and control plants after inoculation with *P. capsici*. Photographs were taken after inoculation with *P. capsici* three days. The diameter of the plug of *P. capsici* is 0.5cm. The red dotted line was used to label the lesion area in each leaf. **(B)** Percentage of the lesion area of the leaves three days after inoculation with *P. capsici*. Bars with different lower case letters indicate significant differences at P ≤ 0.05. Mean values and SDs for three biological replicates are shown.

Forty-five days after injection, detached leaves of plants silenced for each of the fifteen CaSBP genes and those of the negative control plants were inoculated with P. capsici strain HX-9. Three days post-inoculation with HX-9, the detached leaves of the negative control plants exhibited large hygrophanous lesions, which occupied almost 80% of the whole leaf area (**Figure 2A**). Additionally, the detached leaves of the plants silenced for CaSBP01, CaSBP02, CaSBP03, CaSBP04, CaSBP05, CaSBP06, CaSBP07, CaSBP09, CaSBP10, CaSBP14, and CaSBP15 also exhibited large hygrophanous lesions without any noticeable difference from the negative control (**Figures 2A, B**). However, the detached leaves of the CaSBP08-, CaSBP11-, CaSBP12-, and CaSBP13-silenced plants exhibited smaller hygrophanous lesions or lacked them altogether (**Figure 2A**). In addition, the percentages of lesion areas on the leaves of CaSBP08-, CaSBP11-, CaSBP12-, and CaSBP13-silenced plants and negative control plants also significantly differed (**Figure 2B**). Detailed statistics of the disease incidence of detached leaves from CaSBP-silenced and negative control plants are available in **Table S4**. These results indicated that four CaSBP genes (CaSBP08, CaSBP11, CaSBP12, and CaSBP13) responded to P. capsici infection in pepper.

### *CaSBP08* Protein Localization in the Nucleus

**Figure 3:**
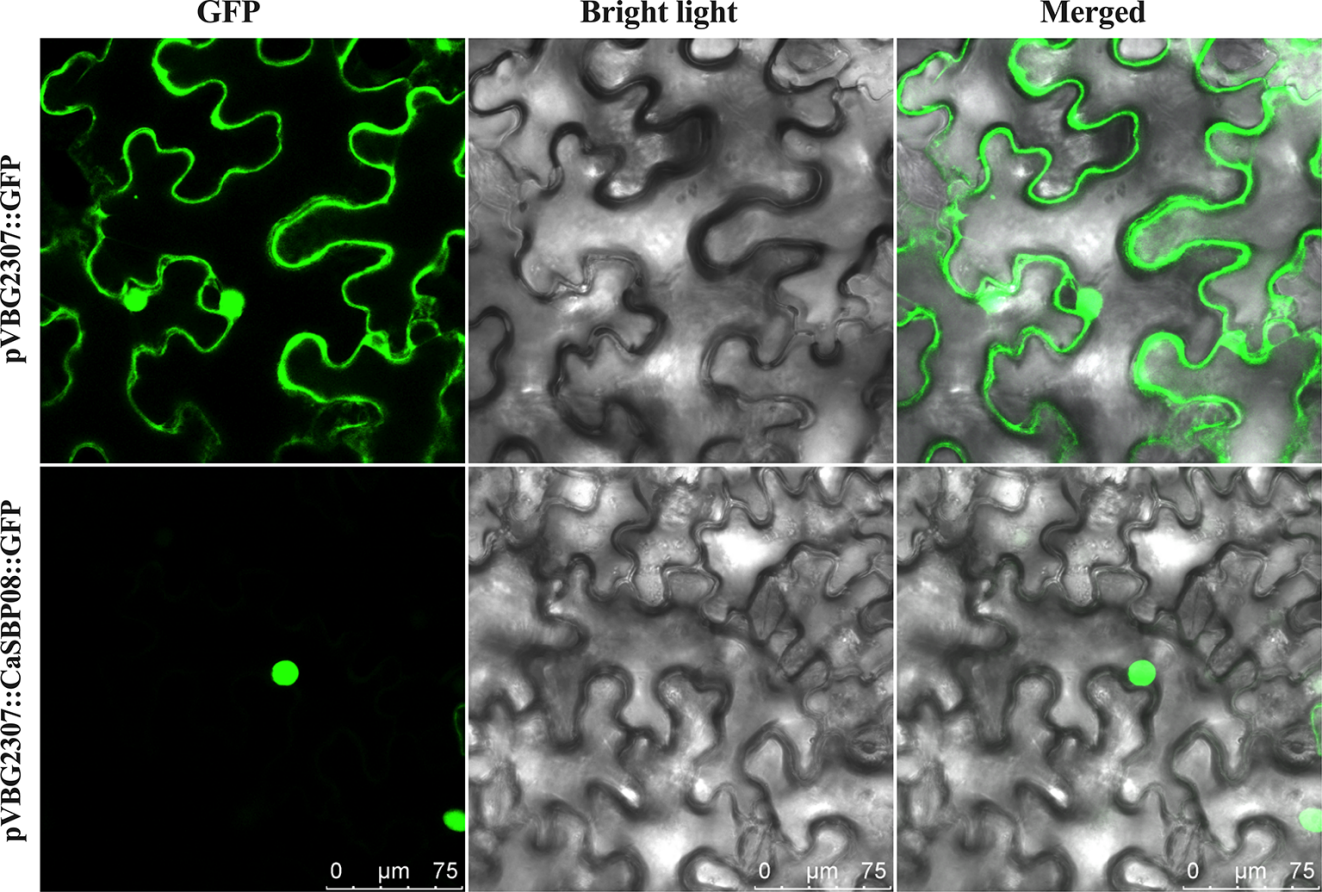
Subcellular localization of the CaSBP08 protein. *Agrobacterium tumefaciens* strain GV3101 with pVBG2307:*CaSBP08*:*GFP* and pVBG : *GFP* (used as a control) vectors were transiently expressed in *N. benthamiana* leaves. The fluorescence was visualized using a laser scanning confocal microscope under bright and fluorescent fields. The photographs were taken in a dark field for green fluorescence and under bright light for the morphology of the cell. Bars in this picture are 75μm.

To determine the subcellular localization of CaSBP08 protein, the GV3101 strain of *A. tumefaciens* with pVBG2307:*CaSBP08*:*GFP* and pVBG2307:*GFP* (used as a control) vectors were rapidly expressed in the leaves of *N. benthamiana* plants. The results indicated that the control (pVBG2307:*GFP*) exhibited GFP signals in the whole cell, including the nucleus, cytoplasm, cell wall, and cell membrane, whereas, pVBG2307:*CaSBP08*:*GFP* only exhibited GFP signals in the nucleus (**Figure 3**). This indicated that CaSBP08 protein was localized in the nucleus.

### Silencing of *CaSBP08* Enhanced Pepper Resistance to *P. capsici* Infection

**Figure 4:**
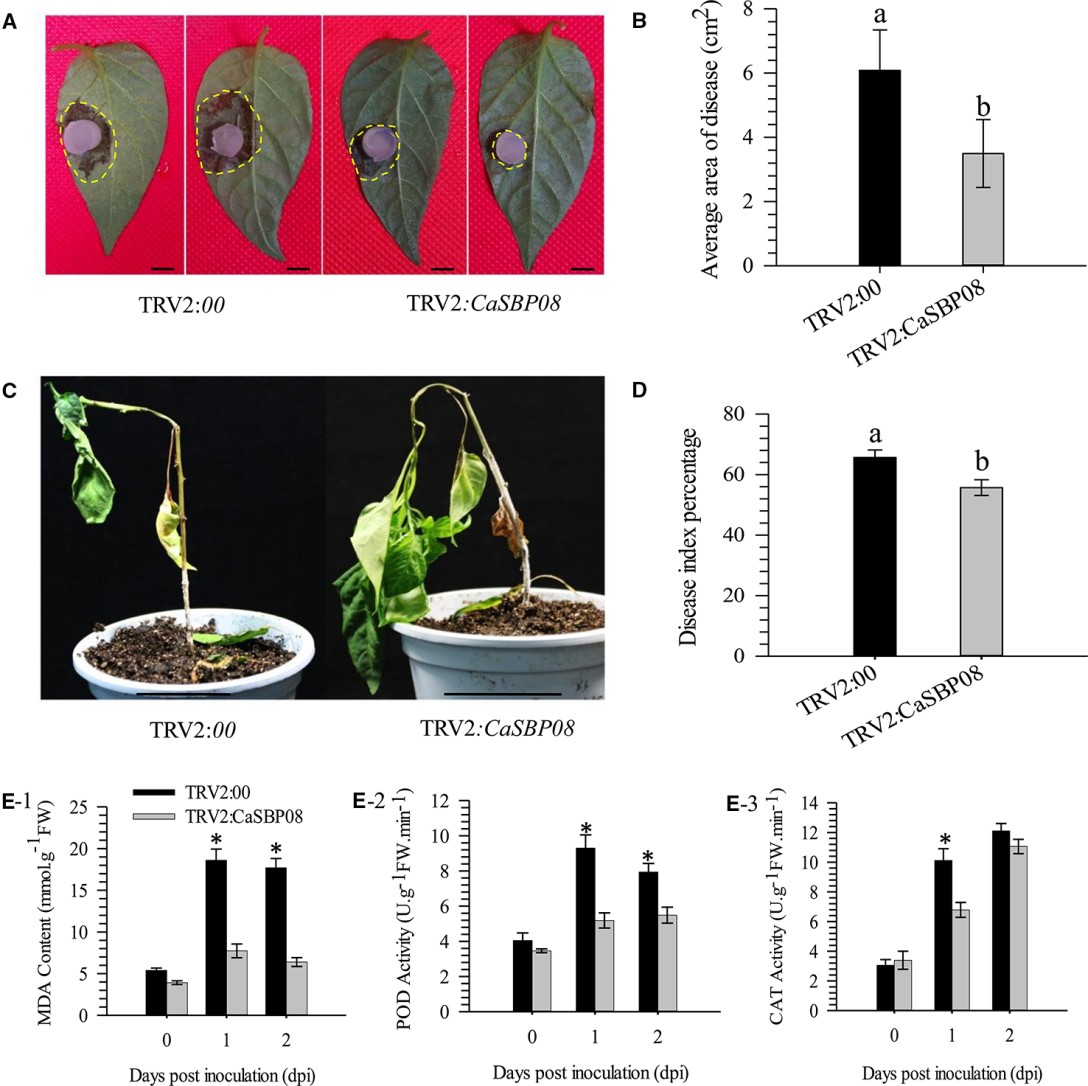
Loss function analysis of *CaSBP08* in pepper plant defense response against *P. capsici* infection. **(A)** Phenotypes of detached leaves of *CaSBP08* silenced and negative control plants after inoculation with *P. capsici*. Photographs were taken at two days after inoculation with *P. capsici*. The yellow dotted line was used to label the lesion area in each leaf. **(B)** The average diseased areas of the detached leaves of the *CaSBP08* silenced and negative control plants. Data were collected, two days after inoculation with *P. capsici*. **(C)** Phenotypes of the *CaSBP08* silenced and negative control plants after inoculation with *P. capsici* sixteen days. **(D)** The disease index percentage of the *CaSBP08* silenced and negative control plants and data were collected sixteen days after inoculation with *P. capsici*. **(E)** Determination of MDA content **(E-1)**, POD activity **(E-2)**, and CAT activity **(E-3)** of *CaSBP08* silenced and negative control plants after inoculating with *P. capsici*. Bars in Figure A are 0.5cm, and C are 4.5cm. Bars with different letters indicate significant differences at P ≤ 0.05. * Represent significant differences at P ≤ 0.05. Mean values and SDs for three biological replicates are shown.

**Figure 5:**
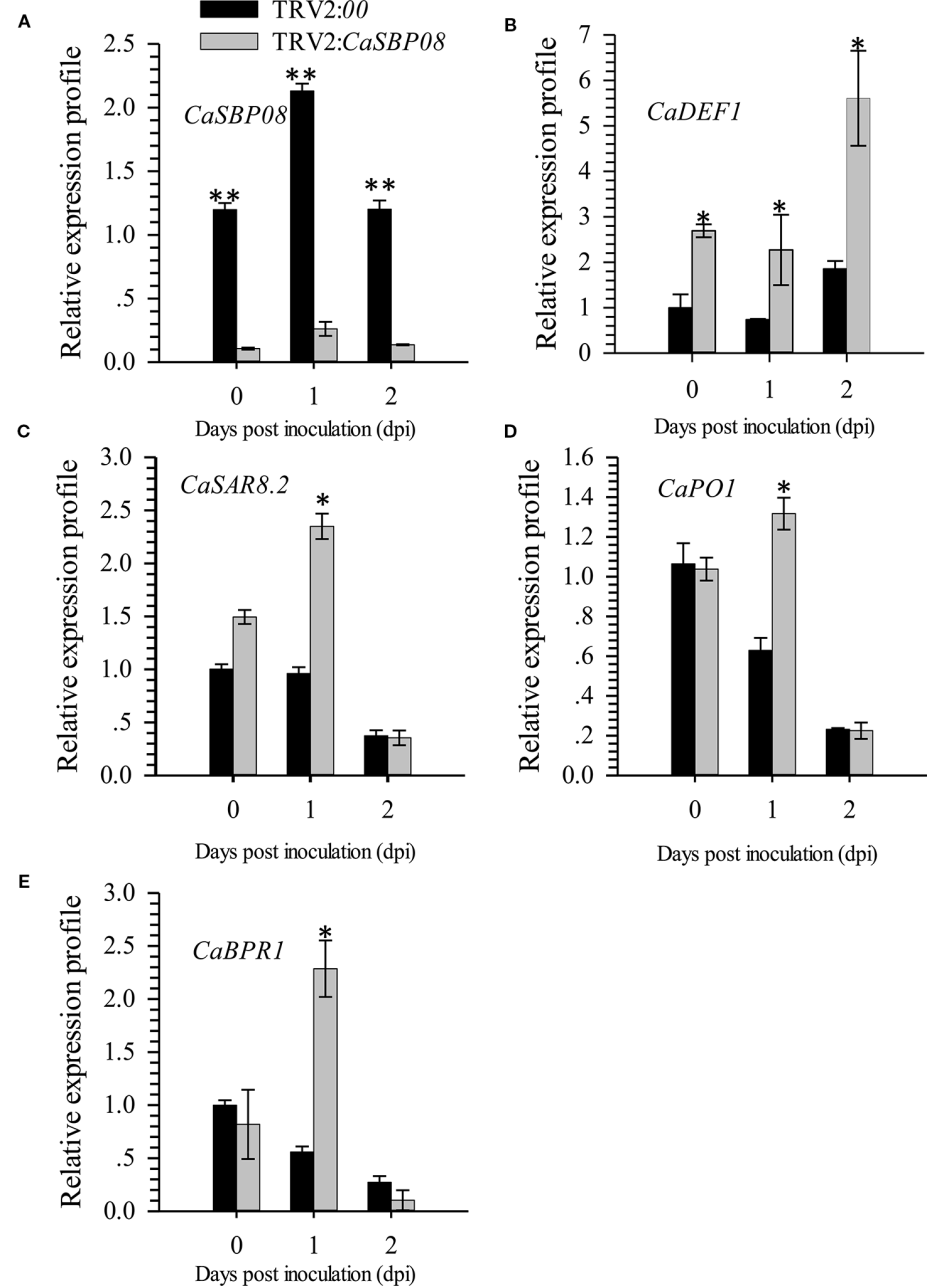
The expression of *CaSBP08* **(A)**, and defense-related genes*, CaDEF1* **(B)**, *CaSAR8.2* **(C)**, *CaPO1* **(D)**, and *CaBPR1* **(E)** after inoculation with *P. capsici* in *CaSBP08* silenced and negative control plants. * and ** represent significant differences at P ≤ 0.05 and P ≤ 0.01 respectively. Mean values and SDs for three biological replicates are shown.

In order to confirm the CaSBP genes involved in resistance to *P. capsici* infection, we first selected CaSBP08 for further study using the virus-induced silencing procedure. As **Figure S1** shows, the CaSBP08 gene was considerably silenced, and the silencing efficiency was 75%. Two days after inoculation with the P. capsici HX-9 strain, the detached leaves of negative control plants showed obvious hygrophanous lesions, while those of the CaSBP08-silenced plants displayed no or small hygrophanous lesions (**Figure 4A**). Additionally, the average diseased area of the leaves of CaSBP08-silenced plants was significantly smaller than that of the negative control plants (**Figure 4B**). Additionally, sixteen days after P. capsici infection, the damage to CaSBP08-silenced plants was less than that to control plants (**Figure 4C**). The disease index percentages of CaSBP08-silenced plants were substantially lower than those for the control plants (**Figure 4D**). Moreover, the MDA content in the CaSBP08-silenced plant was lower than that of the control plants (**Figure 4E-1**). The POD and CAT activities increased in the CaSBP08-silenced and negative control plants (**Figures 4E-2, 4E-3**). However, the POD and CAT activities in the CaSBP08-silenced plants were less than those in the negative control plants (**Figures 4E-2, 4E-3**). The CaSBP08 expression level increased in the beginning and then decreased, but the expression level in the silenced plants was considerably lesser than that in the negative control plants (**Figure 5A**). The expression of the defense genes (CaDEF1, AF442388; CaSAR8.2, AF112868; CaPO1, AF442386; CaBPR1, AF053343) increased in CaSBP08-silenced plants to different degrees, and the expression level was more than that in the negative control plants at day one (**Figures 5B–E**). However, the expression of the defense-related genes (i.e., CaPO1, CaDEF1, CaBPR1, and CaSAR8.2) in the CaSBP08-silenced and negative control plants decreased at day two (except for CaDEF1; **Figures 5B–E**). The results revealed that CaSBP08 played a negative role in the plant defense response against P. capsici infection.

### Overexpression of *CaSBP08* in *N. benthamiana* Increased Susceptibility to *P. capsici* Infection

**Figure 6:**
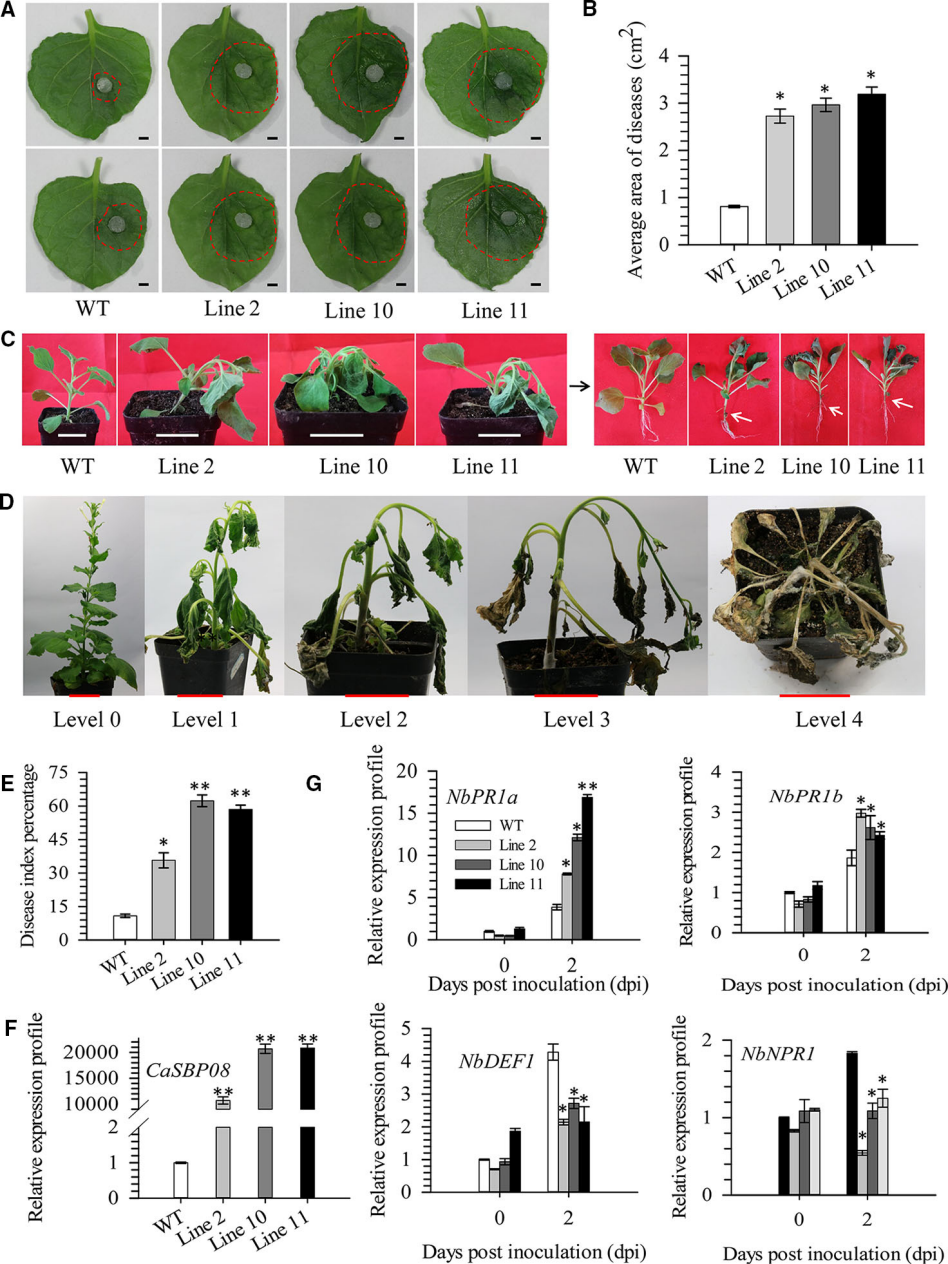
Function analysis of overexpression of *CaSBP08* transgenic lines in defense response against *P. capsici* infection. **(A)** Phenotypes of the detached leaves of transgenic and wild-type plants after inoculation with *P. capsici* two days. The red dotted line was used to label the lesion area in each leaf. **(B)** The average diseased areas of the detached leaves of transgenic and wild-type plants. Data were collected, two days after inoculation with *P. capsici*. **(C)** Phenotypes of transgenic and wild-type plants after inoculation with *P. capsici* three days. The black arrow indicates the phenotype of the left transgenic and wild-type plants after removing the matrix. White arrows indicate the constricted area between root and stem. **(D)** Classification of disease index percentage of transgenic and wild-type plants after inoculation with *P. capsici* thirteen days. **(E)** Disease index percentage of transgenic and wild-type plants and data were collected thirteen days after inoculation with *P. capsici.* **(F)** The expression level of *CaSBP08* in transgenic and wild-type plants. **(G)** The expression of defense-related genes in transgenic and wild-type plants after inoculation with *P. capsici.* Bars in Figure A are 0.4cm, C and D are 3.5cm. * and ** represent significant differences at P ≤ 0.05 and P ≤ 0.01 respectively. Mean values and SDs for three biological replicates are shown.

In order to confirm *CaSBP08* is involved in plant resistance to *P. capsici* infection, transgenic *CaSBP08* lines were obtained by *Agrobacterium*-mediated tobacco leaf disc transformation, as the stable transformation of pepper plants remains challenging. Eleven transgenic lines were acquired, and there were no observable differences among their phenotypes. Then, three *CaSBP08* transgenic lines (lines 2, 10, and 11) were randomly selected for disease resistance assays. Seedlings of forty-day-old plants were used for the following experiment. Two days after the *P. capsici* HX-9 strain inoculation, a small hygrophanous lesion area appeared on the detached leaves of wild-type (WT) plants, while the hygrophanous lesion area occupied almost half of the detached leaves from transgenic lines 2, 10, and 11 (**Figure 6A**). Additionally, the average areas of disease of transgenic lines 2, 10, and 11 were significantly higher than that of WT plants (**Figure 6B**). Three days after HX-9 strain inoculation, no disease symptoms were observed in WT plants, whereas in transgenic plants (lines 2, 10, and 11) wilting and constriction at the junction of the root and stem were observed (**Figure 6C**). In addition, the forty-five-day-old seedings were used for the disease index percentage statistics experiment. Thirteen days post-inoculation with *P. capsici* strain HX-9, the disease symptoms in WT and transgenic plants (lines 2, 10, and 11) were categorized into five levels (**Figure 6D**). The disease index percentages of transgenic lines 2, 4, and 11 were substantially higher than that of WT plants (**Figure 6E**). A detailed summary of disease index percentage data is provided in **Table S5**.

The *CaSBP08* expression in transgenic lines 2, 10, and 11 differed from that of WT plants (**Figure 6F**). The expression of defense-related genes, i.e., *NbPR1a* (pathogenesis-related), *NbPR1b* (pathogenesis-related), *NbDEF1* (defensin), and *NbNPR1* (non-expressor pathogenesis-related), were measured. After *P. capsici* inoculation, the expression of *NbPR1a* (JN247448.1) and *NbPR1b* (XM_016587501.1) increased at day two, and its level in transgenic lines was more than that in the WT plants (**Figure 6G**). The expression levels of *NbDEF1* (X99403) and *NbNPR1* (AF480488) in WT plants increased at day two and were higher than that in the transgenic lines (**Figure 6G**). However, the expression levels of *NbDEF1* and *NbNPR1* in transgenic lines had no obvious changes between day one and day two. These results reveal that *CaSBP08* has a negative role in the defense response of plants against *P. capsici* infection.

## Discussion

The SBP-box gene family is comprised only of plant transcription factors. Gene family memebers participate in different pathways, including those related to plant morphogenesis, floral transition, male sterility, biosynthesis of gibberellic acid (GA), transition from the vegetative to reproductive stage, endoplasmic reticulum (ER) stress signaling, and environmental stress responses (Cardon et al., 1997; Zhang et al., 2007; Shikata et al., 2012; Ning et al., 2017; Zhang J. et al., 2017). These roles of SBP-box genes were investigated in other plants. However, the function of CaSBPs, especially in plant defense responses against P. capsici infection have not yet been studied.

We screened a gene (*CaSBP05*) identified as differentially expressed from our previously established transcriptome database of *P. capsici* across different affinity races. We analyzed the expression patterns of *CaSBP* genes under infections with *P. capsici*-compatible and -incompatible strains and hormonal treatment in our previous study (Zhang et al., 2016). We found that most *CaSBP* genes are induced by hormones and *P. capsici* infection, but there is no direct evidence proving that they are involved in pepper defence mechanisms against *P. capsici* infection (Zhang et al., 2016). Therefore, to screen *P. capsici* infection response genes from among *CaSBP* genes, fifteen *CaSBP* genes were silenced. The phenotypes of the plants with each of the fifteen silenced *CaSBP* genes exhibited no obvious differences compared with the control plants (**Figure 1A**). It is known that most of the genes in SBP-box family are related to plant growth, development, and morphogenesis. For example, overexpression of the small RNA molecule miR156/157, whose target is the protein-regulated SBP-box genes *TfLFY* and *TfMIR172* in *Torenia* plants, can induce bushy plant architectures in *Torenia fournieri* (Shikata et al., 2012). Overexpression of tae-miR156, whose target is the squamosa promoter binding protein-like genes (*TaSPL3/17*) in the bread wheat cultivar ‘Kenong199,' leads to an increase in tiller number and serious flaws in spikelet development, and tae-miR156 mediated suppression of some squamosa promoter binding genes (Liu et al., 2017). *Arabidopsis AtSPL14* mutants with a T-DNA insertion in their squamosa binding protein (SBP) domain exhibited altered architectures with petiole elongation and more serrated leaf margins (Stone et al., 2005). However, these phenomena were not observed in pepper plants with each of the fifteen *CaSBP* genes silenced. Moreover, in order to confirm the silencing specificity of each of the *CaSBP* genes, we also measured the relative expression of genes with the highest homology compared to each of the silent genes.

When the target pepper SBP-box genes were silenced, the expression levels of the pepper SBP-box genes and their corresponding genes with the highest homology can be divided into three categories (**Figure 1C**). In the first, the target gene was silenced (i.e., *CaSBP02* or *CaSBP09*), and the genes with the highest homology (i.e., *CaSBP06* or *CaSBP15*) were also silenced, but with a lower silencing efficiency. The second category included target genes that were silenced (i.e., *CaSBP04*, *CaSBP11*, *CaSBP12*, *CaSBP14*, or *CaSBP15*), while their corresponding genes with highest homology (i.e., *CaSBP12*, *CaSBP09*, *CaSBP04*, or *CaSBP09*) showed increased expression. The third category included target genes that were silenced (i.e., *CaSBP05* or *CaSBP10*), with their genes of highest homology (i.e., *CaSBP10* or *CaSBP05*) remaining unchanged (**Figure 1C**).

Among the fifteen *CaSBP* genes, two genes (*CaSBP02* and *CaSBP06*) contained ankyrin repeats that are present in proteins with different biological roles and are involved in interactions between proteins (Zhang et al., 1999). Thus, there may be a functional relationship between the *CaSBP* genes. The detached leaves of the fifteen different *CaSBP*-silenced plants and the negative control plants were inoculated with the *P. capsici* HX-9 strain. After three days, the detached leaves of the *CaSBP01*-, *CaSBP02*-, *CaSBP03*-, *CaSBP04*-, *CaSBP05*-, *CaSBP06*-, *CaSBP07*-, *CaSBP09*-, *CaSBP10*-, *CaSBP14*-, and *CaSBP15*-silenced plants exhibited large hygrophanous lesions with no difference compared to the negative control (**Figure 2**). Additionally, the detached leaves of the *CaSBP08*-, *CaSBP11*-, *CaSBP12*-, and *CaSBP13*-silenced plants exhibited very small or no hygrophanous lesions, and the percentage of lesion area of the plants silenced for these genes exhibited a significant difference compared with the negative control (**Figure 2**). Therefore, we screened four *CaSBP* genes (*CaSBP08*, *CaSBP11*, *CaSBP12*, and *CaSBP13*) that are involved in plant defense responses to *P. capsici* infection. To further study the function of *CaSBP* genes in the process of *P. capsici* infection response, we chose one of our screened peppers SBP-box gene (*CaSBP08*) for further research.

*CaSBP08* has a 336-bp open reading frame, encoding 111 amino acids (Zhang et al., 2016). The CaSBP08 protein was localized to the nucleus (**Figure 3**). Silencing of this gene enhanced resistance to *P. capsici* infection in pepper plants.

After *P. capsici* inoculation, the lesion areas of detached leaves of *CaSBP08*-silenced plants were smaller than the lesion areas of the negative control plants (**Figures 4A, B**). The disease index percentage of *CaSBP08*-silenced plants was also lower than that of the negative control plants (**Figure 4D**). Furthermore, after inoculation with *P. capsici* using the root-drench method, the MDA content as well as the POD and CAT activities of *CaSBP08*-silenced plants increased but were lower than those of the negative control treatment plants (**Figure 4E**). A plant under stress is closely related to membrane lipid peroxidation, which is induced by active oxygen accumulation. MDA is one of the most important products of membrane lipid peroxidation. Therefore, the degree of membrane lipid peroxidation can be determined by measuring MDA content. This can reflect the degree of damage to the membrane system and the resistance of plants (Ma et al., 2013). Moreover, it has been reported that during *Phytophthora* root rot development there is a relationship between the disease induced by *P. capsici* and the antioxidant system (Koç and Üstün, 2012). The protein encoded by the peroxidase *CanPOD* gene plays a positive role in plant defense responses to *P. capsici* infection in pepper plants (Wang et al., 2013b), as *CanPOD* is related to reactive oxygen species (ROS)-scavenging enzymes. CAT is also a major ROS-scavenging enzyme in plants (Mittler et al., 2004). POD and CAT activity levels were increased during *P*. *capsici* infection in pepper plants (Koç and Üstün, 2012). The high POD activity indicates that pepper plants are extra sensitive to infection by *P. capsici* as POD-mediated enzymatic reactions are enhanced in infected plants (Wu et al., 2016). POD activity increase is an essential factor in enhancing resistance to plant disease (Wu et al., 2016). The activity of POD increases during the first stage of *Xanthomonas campestris* pv*. vesicatoria* infection and then declines when the H_2_O_2_ accumulation reaches its maximum in pepper plants (Do et al., 2003). After *P. capsici* inoculation, the increased rate of POD activity in susceptible varieties was greater than that in resistant varieties of Kernel Pumpkin (Zhou et al., 2003). Moreover, catalase plays a key role in maintaining H_2_O_2_ homeostasis in cells and has been implicated in ROS signaling in response to pathogen attack (Magbanua et al., 2007). In addition, during infection, *Phytophthora nicotianae* increases its own peroxisomal catalase levels while concurrently down-regulating host catalase expression (Blackman and Hardham, 2008). The activity of CAT in cucumber varieties resistant to downy mildew was lower compared with susceptible varieties (Wang, 2001).

It has been reported that the strong suppression of pepper *CaPO1* can cause dramatic H_2_O_2_ accumulation and a huge decrease in peroxidase activity during programmed cell death (Do et al., 2003). Therefore, we detected the expression level of *CaPO1* subsequent to *P. capsici* inoculation into pepper plants. The level of *CaPO1* expression was suppressed in the negative control plants and first increased and then decreased in the *CaSBP08*-silenced plants (**Figure 5**). Bae et al. (2006) reported that poplar *PoPOD1* is suppressed under NaCl, methyl viologen, polyethylene glycol, gibberellic acid (GA3) and jasmonic acid (JA) treatments.

Furthermore, it has been reported that SA- and JA-mediated signal transduction pathways play a crucial role in plant resistance to diseases (Spoel et al., 2003). Most *CaSBP* genes can be induced under SA and MeJA treatment and inhibited during the early stage by SA synthesis inhibitor (paclobutrazol, PBZ) and MeJA (salicylhydroxamic acid, SHAM) synthesis inhibitor treatments (Zhang et al., 2016). To determine whether *CaSBP08* is involved in the SA- and MeJA-mediated resistant pathways, we also studied and detected the expression of some defense-related genes. For example, the molecular marker *CaSAR8.2* can be used for the detection of several pathogenic diseases that affect the SA-mediated signal transduction pathway (Lee and Hwang, 2003). *CaBPR1* is involved in the hypersensitive response and was induced in an incompatible interaction of leaves with *Xanthomonas campestris* pv. *vesicatoria* (Kim and Hwang, 2000). *CaDEF1* is involved in the MeJA-mediated signal transduction pathway, which has functions in microbial infection, as abiotic elicitors, and in response to some environmental stressors (Do et al., 2004). In this work, the *CaSAR8.2* and *CaBPR1* expression levels were induced at the first stage and then decreased in *CaSBP08*-silenced plants, while they were suppressed in the negative control plants (**Figure 5**). Furthermore, the expression level of *CaDEF1* increased in negative control plants and *CaSBP08*-silenced plants and was higher in *CaSBP08*-silenced plants compared with negative control plants. The results demonstrate the involvement of *CaSBP08* in the SA- and MeJA-mediated resistant pathways. Further research, however, is need in this regard.

Ectopic expression of *BpSPL9* in the *Betula platyphylla* Suk. (birch) has been reported to enhance the ROS scavenging under drought and salt stress (Ning et al., 2017). *SPL9* interacts with JA ZIM-domain (JAZ) proteins and negatively regulates JA response, though it promotes JAZ3 accumulation in *Arabidopsis* (Mao et al., 2017). Similarly, *VpSBP5* likely participates in regulating resistance against *Erysiphe necator* though SA- and MeJA-mediated signal transduction pathway in grapes (Hou et al., 2013). Overexpression of *CaSBP08* in transgenic *N. benthamiana* enhanced susceptibility to *P. capsici* infection, as demonstrated by the higher average disease area and disease index percentage compared to WT plants (**Figures 6B, E**). Besides, the *NbPR1a* and *NbPR1b* genes, which are involved in the SA-induced systemic acquired resistance pathway and JA-mediated disease resistance signaling pathway, respectively, were induced in the *CaSBP08* transgenic and WT plants (Sohn et al., 2007; Cheol Song et al., 2016). *PR1a* and *PR1b*, two reported defense-related genes in tobacco, can be highly induced by tobacco mosaic virus (TMV) (van Huijsduijnen et al., 1985). The expression level of the SA signaling marker gene *NbPR1a* was associated with systemic acquired resistance (SAR) against *Pseudomonas syringae* pv. *tabaci* (Cheol Song et al., 2016). *PR1b* is a JA-responsive gene in tobacco (Sohn et al., 2007). Moreover, it has been reported that ectopic overexpression of *CaC3H14* enhances resistance of tobacco to *Ralstonia solanacearum* infection, and the expression of *PR1b* was induced in transgenic and WT plants. However, the expression of *PR1b* in transgenic lines was significantly lower than that in WT plants (Qiu et al., 2018). The results of the present study suggest that *CaSBP08* may be involved in resistance to *P. capsici* through regulating the expression of defense-related genes. However, further research is need to examine the regulatory mechanism. In *N. benthamiana*, *SPL6* plays a positive regulatory role in nucleotide binding rich leucine repeat (N TIR-NB-LRR) receptor-mediated plant natural immunity (Padmanabhan et al., 2013). Overexpression of *JcNAC1* in *Jatropha curcas* can enhance susceptibility of plants to *Botrytis cinerea* infection and inhibit the expression of some defense-related marker genes (Qin et al., 2014). Overexpression of *ATAF2* in *Arabidopsis* can enhance the sensitivity of plants to *Fusarium oxysporum* infection and repress the expression of pathogenesis-related genes (Delessert et al., 2005).

## Conclusions

In conclusion, we screened four genes (*CaSBP08*, *CaSBP11*, *CaSBP12*, and *CaSBP13*) out of the fifteen identified *CaSBP* genes, each of which responded to *P. capsici* infection. Additionally, we selected one of our screened pepper SBP-box genes (*CaSBP08*) for further research. CaSBP08 protein was thus observed to be localized to the nucleus. Silencing *CaSBP08* enhanced resistance against *P. capsici* infection, such that the average disease area, the percent disease index, and the POD and CAT activities were lower in the *CaSBP08*-silenced plants compared with the negative control plants. Additionally, following inoculation with *P. capsici*, the defense genes *CaPO1*, *CaDEF1*, *CaBPR1*, and *CaSAR8.2* were induced during the early stage of infection in *CaSBP08*-silenced plants, while *CaPO1*, *CaBPR1*, and *CaSAR8.2* were suppressed in the negative control plants. In addition, overexpression of *CaSBP08* in *N. benthamiana* enhanced susceptibility to *P. capsici* infection, as demonstrated by the average disease area and the percent disease index being greater than those of WT plants. Our work provides a basis for future research on the role of *CaSBP* genes in plant resistance to infections by *P. capsici* and similar pathogens.

## Data Availability Statement

All datasets generated for this study are included in the article/**Supplementary Material**.

## Author Contributions

H-XZ and Z-HG perceived and designed research. H-XZ, X-HF, and J-HJ carried out the experiments. H-XZ analyzed data. H-XZ wrote the manuscript. MA and AK revised the manuscript. Z-HG and A-MW contributed reagents/materials/analysis tools. All authors read and approved the manuscript.

## Conflict of Interest

The authors declare that the research was conducted in the absence of any commercial or financial relationships that could be construed as a potential conflict of interest.
