# Supplementary material for: Identification of Pepper CaSBP08 Gene in Defense Response Against Phytophthora capsici Infection
Source: Front Plant Sci. 2020 Feb 26;11:183. doi: 10.3389/fpls.2020.00183 (PMC7054287; doi:10.3389/fpls.2020.00183)
Supplement: Supplementary file 1 [file DataSheet_1.docx]

**Table S1.** Primers names and their sequences used for vector construction in this study.

| Olig name | Primer Abbreviation | Primer Sequence (5^’-^3^’^) |
| --- | --- | --- |
| *CaSBP01* | CaSBP01-VIGS-F | CGGGATCCAGTGGTGGTTATGGTTCTGATGTGA |
|  | CaSBP01-VIGS-R | GGGGTACCCCAATGGTCTCACAGATCCGCT |
| *CaSBP02* | CaSBP02-VIGS-F | CGGGATCCAGGAGAAAGACACAATCGGAAT |
|  | CaSBP02-VIGS-R | GGGGTACCTGACCTGTTGTTCAGCAAGTTT |
| *CaSBP03* | CaSBP03-VIGS-F | CGGGATCCATGTTGGATGTGGCGTGGTTAC |
|  | CaSBP03-VIGS-R | GGGGTACCGATTTGACTAGAAGTATTTGCCTCC |
| *CaSBP04* | CaSBP04-VIGS-F | CGGGATCCCGAATAATACACACGAAACTTCCCTAC |
|  | CaSBP04-VIGS-R | GGGGTACCGTGCCGTCGGTGGTAGTGTT |
| *CaSBP05* | CaSBP05-VIGS-F | CGGGATCCGAACCATAAATGAAGCCGAAG |
|  | CaSBP05-VIGS-R | GGGGTACCGACAGAATCGCTGCTGGAGT |
| *CaSBP06* | CaSBP06-VIGS-F | CGGGATCCGAATGATTGGGTATGGGATG |
|  | CaSBP06-VIGS-R | GGGGTACCTGAAACCTACTACACTGCTGAC |
| *CaSBP07* | CaSBP07-VIGS-F | CGGGATCCAACAACTAATAACCAGCTGAAATCC |
|  | CaSBP07-VIGS-R | GGGGTACCCTTTCGTCGCCTTTCGTTGT |
| *CaSBP08* | CaSBP08-VIGS-F | CGGGATCCGAAGATGAAACTGTATTAATTGGAAAG |
|  | CaSBP08-VIGS-R | GGGGTACCCATTGTTGGCAAAACCTGTG |
| *CaSBP09* | CaSBP09-VIGS-F | CGGGATCCTTTTGGTTCATTGGTAGGTGTATTT |
|  | CaSBP09-VIGS-R | GGGGTACCACACAAGTAGTGCCTGCTGTATTC |
| *CaSBP10* | CaSBP10-VIGS-F | CGGGATCCGAAAATAGCAAATGGGAAGGGAAG |
|  | CaSBP10-VIGS-R | GGGGTACCGACGGCGATGGTATGCCTTAG |
| *CaSBP11* | CaSBP11-VIGS-F | CGGGATCCAGGACTGCCTGCCGTAACAAC |
|  | CaSBP11-VIGS-R | GGGGTACCGACGAGCCCTGTGATTGAGATG |
| *CaSBP12* | CaSBP12-VIGS-F | CGGGATCCATCCTCCGTTATGCTTTCTGGC |
|  | CaSBP12-VIGS-R | GGGGTACCTACCTTGGGAATGGGTGAAACA |
| *CaSBP13* | CaSBP13-VIGS-F | CGGGATCCTGTTTTCCTGGAGTCTCAGATTC |
|  | CaSBP13-VIGS-R | GGGGTACCCATCGCCATGCTGAGCTATC |
| *CaSBP14* | CaSBP14-VIGS-F | CGGGATCCCTATCATCGGGTTCTATGTTTACG |
|  | CaSBP14-VIGS-R | GGGGTACCTGATTATTCTTGCCGTCCCTAT |
| *CaSBP15* | CaSBP15-VIGS-F | CGGGATCCCATTTTTGGTTCAGTGGTAGGTG |
|  | CaSBP15-VIGS-R | GGGGTACCAGACTGAATTCTGCCTTGGTAGA |
| *CaSBP08* | CaSBP08-2307-GFP-F | GCTCTAGAATGGCAACCCAAATCTATGGTAG |
|  | CaSBP08-2307-GFP-R | GGGGTACCAAAATAATGAGATGACATAGTTTTGTTTAG |

**Table S2.** Primers names and their sequences used in this study for quantitative real-time PCR.

| **Olig name** | **Primer Abbreviation** | **Primer Sequence (5^’-^3^’^)** |
| --- | --- | --- |
| ***CaSBP01*** | RTCaSBP01-VIGS-F | GGGAGTTGAAGAAGAGGGAG |
|  | RTCaSBP01-VIGS-R | CCAATGGTCTCACAGATCCG |
| ***CaSBP02*** | RTCaSBP02-VIGS-F | CTCCAACGGAACAAACCCCAC |
|  | RTCaSBP02-VIGS-R | GAGGTGCCTGTGAACCATTTGAG |
| ***CaSBP03*** | RTCaSBP03-VIGS-F | GCTGGCAAGCCTATGGAGTT |
|  | RTCaSBP03-VIGS-R | TAACCACGCCACATCCAACA |
| ***CaSBP04*** | RTCaSBP04-VIGS-F | ATGACAAATTATCAACACCAGCA |
|  | RTCaSBP04-VIGS-R | AAGCAAAATACGTTCTCCCG |
| ***CaSBP05*** | RTCaSBP05-VIGS-F | ATGGACACTAACAAATGGGAAGG |
|  | RTCaSBP05-VIGS-R | GATGTGATGACCCTCCACCAGA |
| ***CaSBP06*** | RTCaSBP06-VIGS-F | TTTGGCATACTGGATGGGTTTA |
|  | RTCaSBP06-VIGS-R | CCTTCCAGGGCACATAATAACC |
| ***CaSBP07*** | RTCaSBP07-VIGS-F | TGCTAAGCAGTACCATAAGAGGC |
|  | RTCaSBP07-VIGS-R | CAACTTGACCACACATTGTCACT |
| ***CaSBP08*** | RTCaSBP08-VIGS-F | ATGGCAACCCAAATCTATGGT |
|  | RTCaSBP08-VIGS-R | TATCACTCTTTTCCCCCTTTTTCTT |
| ***CaSBP09*** | RTCaSBP09-VIGS-F | TTTTGGTTCATTGGTAGGTGTATTT |
|  | RTCaSBP09-VIGS-R | TTGCCATCATCAAACTCATCCA |
| ***CaSBP10*** | RTCaSBP10-VIGS-F | GGACGACGAGGAAGACGAAGAT |
|  | RTCaSBP10-VIGS-R | TCGAACTCCGCCAACTGATGAA |
| ***CaSBP11*** | RTCaSBP11-VIGS-F | CATCTCAATCACAGGGCTCG |
|  | RTCaSBP11-VIGS-R | CATTACTATCCTGCTTCACTTGC |
| ***CaSBP12*** | RTCaSBP12-VIGS-F | GTTTCACCCATTCCCAAGGTAATT |
|  | RTCaSBP12-VIGS-R | TAGTACGTCGGTAAAGTCGATTAACAA |
| ***CaSBP13*** | RTCaSBP13-VIGS-F | ATCTCTTTTCTCTACACACTATGGG |
|  | RTCaSBP13-VIGS-R | GAATCTGAGACTCCAGGAAAACA |
| ***CaSBP14*** | RTCaSBP14-VIGS-F | AGAAATAACTGAAAGCACGGAG |
|  | RTCaSBP14-VIGS-R | TGATTATTCTTGCCGTCCCTAT |
| ***CaSBP15*** | RTCaSBP15-VIGS-F | GTCGGGTTCAATTTCTACAGACTCAT |
|  | RTCaSBP15-VIGS-R | CAGCCAGTAGATGAAACCTAAGTAAAGTAT |
| ***CaActin2*** | CaActin2-F | TCCACCTCTTCACTCTCTGCTC |
|  | CaActin2-R | TGACCCATCCCTACCATAACAC |
| ***CaPO1*** | CaPO1-F | GGCGCCAGGATTGCTGACAA |
|  | CaPO1-R | GTGGACATAATCCTCGAAGC |
| ***CaDEF1*** | CaDEF-F | CAAGGGAGTATGTGCTAGTGAGAC |
|  | CaDEF-R | TGCACAGCACTATCATTGCATAC |
| ***CaSAR8.2*** | CaSAR8.2-F | CAGGGAGATGAATTCTGAGGC |
|  | CaSAR8.2-R | CATATGAACCTCTATGGATTTCTG |
| ***CaBPR1*** | CaBPR1-F | CAGGATGCAACACTCTGGTGG |
|  | CaBPR1-R | ATCAAAGGCCGGTTGGTC |
| ***Nbactin-97*** | Nbactin-F | TATGGAAACATTGTGCTCAGTGG |
|  | Nbactin-R | CCAGATTCGTCATACTCTGCC |
| ***NbDEF1*** | NbDEF1RT-F | AACTTGTGAGTCCCAGAG |
|  | NbDEF1RT-R | GGATACCTTTCTACCACC |
| ***NbNPR1*** | NbNPR1-RT-F | TTACTTCACTGAAACGCCT |
|  | NbNPR1-RT-R | CACTTCCTTTAATTCCACCT |
| ***NbPR1a*** | NbPR1a-RT-F | GTAATATCCCACTCTTGCCG |
|  | NbPR1a-RT-R | ATGAAATCGCCACTTCCCTC |
| ***NbPR1b*** | NbPR1b-RT-F | TCAAGCTCAAAACTCTCCCC |
|  | NbPR1b-RT-R | CCACATCTTTACTGCTCCCG |

**Table S3**. Genes with high homology in the pepper SBP-box gene family

| Gene name | The corresponding gene name with high homology |
| --- | --- |
| *CaSBP02* | *CaSBP06* |
| *CaSBP04* | *CaSBP12* |
| *CaSBP05* | *CaSBP10* |
| *CaSBP09* | *CaSBP15* |
| *CaSBP10* | *CaSBP05* |
| *CaSBP11* | *CaSBP09* |
| *CaSBP12* | *CaSBP04* |
| *CaSBP14* | *CaSBP11* |
| *CaSBP15* | *CaSBP09* |

**Table S4.** Statistics on the incidence of detached leaves of pepper SBP-box family genes^,^ silent plant after inoculation with *Phytophthora* *capsici* three days*.*

| Silent plant | Total number of detached leaves | Number of infected leaves | Number of uninfected leaves | Morbidity (%) | Percentage of lesion area to the leaf (%) |
| --- | --- | --- | --- | --- | --- |
| TRV2:*00* | 11 | 9 | 2 | 81.82 | 93.32±5.84a |
| TRV2:*CaSBP01* | 12 | 11 | 1 | 91.67 | 83.11±20.96a |
| TRV2:*CaSBP02* | 10 | 6 | 4 | 60 | 87.17±15.53a |
| TRV2:*CaSBP03* | 10 | 10 | 0 | 100 | 83.29±22.51a |
| TRV2:*CaSBP04* | 10 | 6 | 4 | 60 | 87.07±7.50a |
| TRV2:*CaSBP05* | 7 | 6 | 1 | 85.71 | 81.16±20.31a |
| TRV2:*CaSBP06* | 13 | 11 | 2 | 84.62 | 86.05±5.84a |
| TRV2:*CaSBP07* | 10 | 6 | 4 | 60 | 87.52±8.84a |
| TRV2:*CaSBP08* | 13 | 5 | 8 | 38.46 | 43.78±2.91b |
| TRV2:*CaSBP09* | 8 | 7 | 1 | 87.5 | 89.67±10.83a |
| TRV2:*CaSBP10* | 10 | 6 | 4 | 60 | 84.29±14.82a |
| TRV2:*CaSBP11* | 18 | 2 | 16 | 11.11 | 20.88±9.15b |
| TRV2:*CaSBP12* | 15 | 4 | 11 | 26.67 | 21.43±8.74b |
| TRV2:*CaSBP13* | 10 | 3 | 7 | 30 | 34.63±3.78b |
| TRV2:*CaSBP14* | 10 | 6 | 4 | 60 | 83.34±24.53a |
| TRV2:*CaSBP15* | 9 | 7 | 2 | 77.78 | 86.04±16.30a |

**Figure S1.** The silencing efficiency of *CaSBP08* in the silenced and negative control plants. Bars with different letters indicate significant differences at P ≤ 0.05. Mean values and SDs for three biological replicates are shown.

**
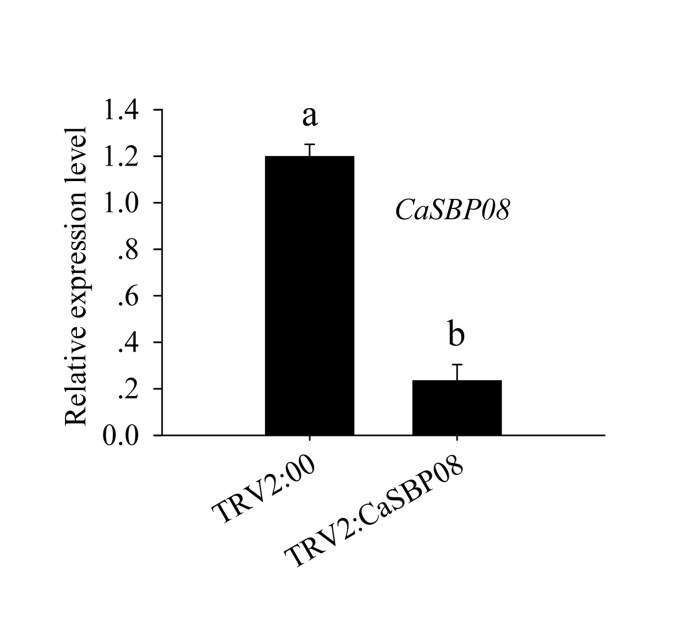
**

**Table S5.** The detail data of disease index percentage of transgenic (line 2, line 10, and line 11) and wild-type plants after inoculated with *P. capsici* thirteen days.

|  | 0 level plants | 1 level plants | 2 level plants | 3 level plants | 4 level plants | Total number of treatment plants | Disease index percentage |
| --- | --- | --- | --- | --- | --- | --- | --- |
| WT | 23 | 0 | 4 | 1 | 0 | 28 | 9.82 |
| Line 2 | 13 | 1 | 1 | 1 | 5 | 21 | 30.95 |
| Line 10 | 5 | 2 | 5 | 2 | 14 | 28 | 66.07 |
| Line 11 | 12 | 0 | 2 | 1 | 15 | 30 | 55.83 |
| WT | 17 | 1 | 1 | 1 | 1 | 21 | 11.9 |
| Line 2 | 18 | 4 | 5 | 6 | 9 | 42 | 40.48 |
| Line 10 | 5 | 3 | 3 | 3 | 9 | 23 | 58.69 |
| Line 11 | 8 | 2 | 2 | 3 | 14 | 29 | 61.21 |
